# *catena*-Poly[[(5,5′-dimethyl-2,2′-bipyridine-κ^2^ *N*,*N*′)cadmium]-di-μ-bromido]

**DOI:** 10.1107/S1600536812023860

**Published:** 2012-05-31

**Authors:** Sadif A. Shirvan, Sara Haydari Dezfuli

**Affiliations:** a Department of Chemistry, Islamic Azad University, Omidieh Branch, Omidieh, Iran

## Abstract

In the crystal of the title polymeric compound, [CdBr_2_(C_12_H_12_N_2_)]_*n*_, the Cd^II^ cation is located on a twofold rotation axis. The Cd^II^ cation is six-coordinated in a distorted octa­hedral geometry formed by two N atoms from the 5,5′-dimethyl-2,2′-bipyridine ligand and four bridging Br^−^ anions. The bridging function of the Br^−^ anions leads to a polymeric chain running along the *c* axis.

## Related literature

For related structures, see: Ahmadi *et al.* (2008 ▶, 2010 ▶); Albada *et al.* (2004 ▶); Amani *et al.* (2007 ▶, 2009 ▶); Han *et al.* (2006 ▶); Kalateh *et al.* (2010 ▶); Karaca *et al.* (2009 ▶); Khalighi *et al.* (2008 ▶); Maheshwari *et al.* (2007 ▶); Tadayon Pour *et al.* (2008 ▶); Zhang (2007 ▶).
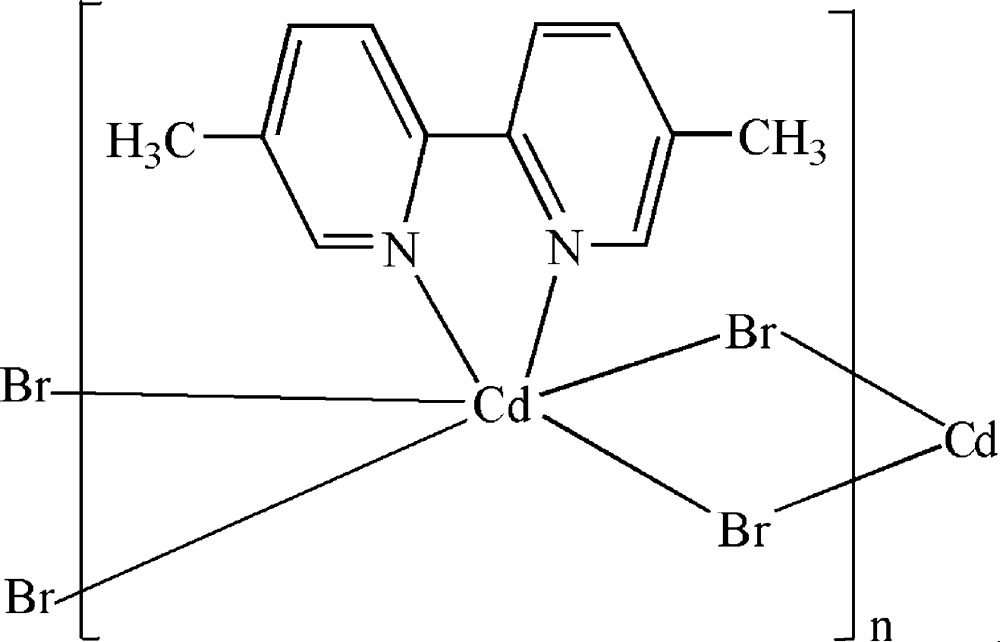


## Experimental

### 

#### Crystal data

[CdBr_2_(C_12_H_12_N_2_)]
*M* *_r_* = 456.45
Monoclinic, 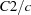

*a* = 19.637 (5) Å
*b* = 9.6563 (15) Å
*c* = 7.485 (2) Å
β = 104.76 (2)°
*V* = 1372.4 (6) Å^3^
*Z* = 4
Mo *K*α radiation
μ = 7.39 mm^−1^
*T* = 298 K
0.12 × 0.11 × 0.09 mm

#### Data collection

Bruker APEXII CCD area-detector diffractometer
Absorption correction: multi-scan (*SADABS*; Bruker, 2001 ▶) *T*_min_ = 0.435, *T*_max_ = 0.548
5378 measured reflections
1346 independent reflections
1015 reflections with *I* > 2σ(*I*)
*R*_int_ = 0.110

#### Refinement

*R*[*F* ^2^ > 2σ(*F* ^2^)] = 0.041
*wR*(*F* ^2^) = 0.091
*S* = 1.03
1346 reflections
78 parameters
H-atom parameters constrained
Δρ_max_ = 0.85 e Å^−3^
Δρ_min_ = −0.70 e Å^−3^

### 

Data collection: *APEX2* (Bruker, 2007 ▶); cell refinement: *SAINT* (Bruker, 2007 ▶); data reduction: *SAINT*; program(s) used to solve structure: *SHELXTL* (Sheldrick, 2008 ▶); program(s) used to refine structure: *SHELXTL*; molecular graphics: *SHELXTL*; software used to prepare material for publication: *SHELXTL*.

## Supplementary Material

Crystal structure: contains datablock(s) I, global. DOI: 10.1107/S1600536812023860/xu5547sup1.cif

Structure factors: contains datablock(s) I. DOI: 10.1107/S1600536812023860/xu5547Isup2.hkl

Additional supplementary materials:  crystallographic information; 3D view; checkCIF report

## Figures and Tables

**Table 1:** Selected bond lengths (Å)

Cd1—N1	2.352 (4)
Cd1—Br1	2.6676 (8)
Cd1—Br1^i^	2.9351 (10)

Symmetry code: (i) 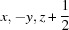.

**Fig. 1.**
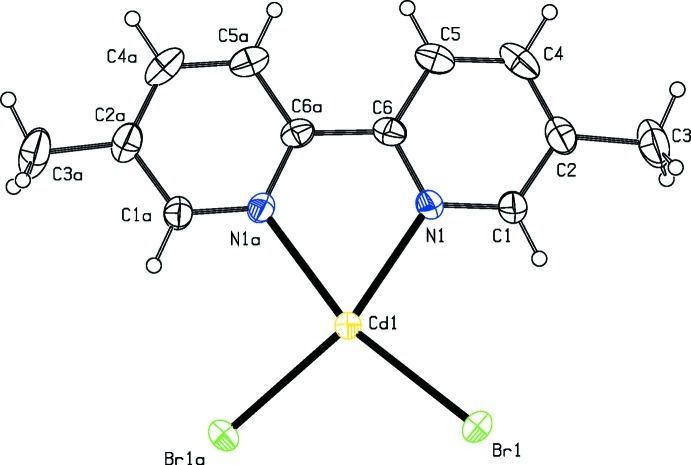
The molecular structure of the title molecule, with the atom-numbering scheme. Displacement ellipsoids are drawn at the 30% probability level [Symmetry codes: (a) 1 - x,y,1/2 - z].

## supplementary crystallographic information

### Comment

5,5'-Dimethyl-2,2'-bipyridine (5,5'-dmbipy), is a good bidentate ligand, and numerous complexes with 5,5'-dmbipy have been prepared, such as that of zinc (Khalighi *et al.*, 2008), indium (Kalateh *et al.*, 2010), iron (Amani *et al.*, 2007), platin (Amani *et al.*, 2009; Maheshwari *et al.*, 2007), copper (Albada *et al.*, 2004), gold (Karaca *et al.*, 2009), cadmium (Ahmadi *et al.*, 2008,2010) and mercury (Tadayon Pour *et al.*, 2008). Here, we report the synthesis and structure of the title compound.

The asymmetric unit of the title compound, (Fig. 1), contains one half-molecule; a twofold rotation axis passes through the Cd atom. The Cd^II^ atom is six-coordinated in a distorted octahedral configuration by two N atoms from 5,5'-dimethyl-2,2'-bipyridine and four bridging Br atoms. The bridging function of the bromide atoms leads to a one-dimensional chain structure. The Cd—Br and Cd—N bond lengths and angles (Table 1) are within normal range [Cd(phen)(µ-Br)_2_]_n_, (Zhang, 2007) and [Cd(bipy)(µ-Br)_2_]_n_, (Han *et al.*, 2006) [where phen is 1,10-phenanthroline and bipy is 2,2'-bipyridine].

### Experimental

For the preparation of the title compound, a solution of 5,5'-dimethyl-2,2'-bipyridine (0.25 g, 1.33 mmol) in methanol (10 ml) was added to a solution of CdBr_2_.4H_2_O (0.46 g, 1.33 mmol) in methanol (10 ml) at room temperature. The suitable crystals for X-ray diffraction experiment were obtained by methanol diffusion to a colorless solution in DMSO. Suitable crystals were isolated after one week (yield; 0.45 g, 74.1%).

### Refinement

H atoms were positioned geometrically with C—H = 0.93 Å and constrained to ride on their parent atoms, with *U*_iso_(H) = 1.2*U*_eq_(C).

### Crystal data

**Table:**

[CdBr_2_(C_12_H_12_N_2_)]	*F*(000) = 864
*M**_r_* = 456.45	*D*_x_ = 2.209 Mg m^−^^3^
Monoclinic, *C*2/*c*	Mo *K*α radiation, λ = 0.71073 Å
*a* = 19.637 (5) Å	Cell parameters from 5378 reflections
*b* = 9.6563 (15) Å	θ = 2.2–26.0°
*c* = 7.485 (2) Å	µ = 7.39 mm^−^^1^
β = 104.76 (2)°	*T* = 298 K
*V* = 1372.4 (6) Å^3^	Prism, colorless
*Z* = 4	0.12 × 0.11 × 0.09 mm

### Data collection

**Table:**

Bruker APEXII CCD area-detector diffractometer	1346 independent reflections
Radiation source: fine-focus sealed tube	1015 reflections with *I* > 2σ(*I*)
Graphite monochromator	*R*_int_ = 0.110
ω scans	θ_max_ = 26.0°, θ_min_ = 2.2°
Absorption correction: multi-scan (*SADABS*; Bruker, 2001)	*h* = −22→24
*T*_min_ = 0.435, *T*_max_ = 0.548	*k* = −10→11
5378 measured reflections	*l* = −9→9

### Refinement

**Table:**

Refinement on *F*^2^	Primary atom site location: structure-invariant direct methods
Least-squares matrix: full	Secondary atom site location: difference Fourier map
*R*[*F*^2^ > 2σ(*F*^2^)] = 0.041	Hydrogen site location: inferred from neighbouring sites
*wR*(*F*^2^) = 0.091	H-atom parameters constrained
*S* = 1.03	*w* = 1/[σ^2^(*F*_o_^2^) + (0.0403*P*)^2^] where *P* = (*F*_o_^2^ + 2*F*_c_^2^)/3
1346 reflections	(Δ/σ)_max_ = 0.004
78 parameters	Δρ_max_ = 0.85 e Å^−^^3^
0 restraints	Δρ_min_ = −0.70 e Å^−^^3^

### Special details

**Table:**

Geometry. All e.s.d.'s (except the e.s.d. in the dihedral angle between two l.s. planes) are estimated using the full covariance matrix. The cell e.s.d.'s are taken into account individually in the estimation of e.s.d.'s in distances, angles and torsion angles; correlations between e.s.d.'s in cell parameters are only used when they are defined by crystal symmetry. An approximate (isotropic) treatment of cell e.s.d.'s is used for estimating e.s.d.'s involving l.s. planes.
Refinement. Refinement of *F*^2^ against ALL reflections. The weighted *R*-factor *wR* and goodness of fit *S* are based on *F*^2^, conventional *R*-factors *R* are based on *F*, with *F* set to zero for negative *F*^2^. The threshold expression of *F*^2^ > σ(*F*^2^) is used only for calculating *R*-factors(gt) *etc*. and is not relevant to the choice of reflections for refinement. *R*-factors based on *F*^2^ are statistically about twice as large as those based on *F*, and *R*- factors based on ALL data will be even larger.

### Fractional atomic coordinates and isotropic or equivalent isotropic displacement parameters (Å^2^)

**Table:**

	*x*	*y*	*z*	*U*_iso_*/*U*_eq_	
C1	0.3751 (3)	0.2708 (6)	−0.0036 (8)	0.0498 (15)	
H1	0.3565	0.1843	−0.0435	0.060*	
C2	0.3367 (3)	0.3894 (7)	−0.0747 (9)	0.0549 (16)	
C3	0.2663 (4)	0.3740 (9)	−0.2097 (11)	0.085 (3)	
H3A	0.2355	0.3221	−0.1537	0.102*	
H3B	0.2718	0.3261	−0.3175	0.102*	
H3C	0.2465	0.4639	−0.2444	0.102*	
C4	0.3660 (3)	0.5147 (7)	−0.0143 (9)	0.0600 (18)	
H4	0.3423	0.5960	−0.0591	0.072*	
C5	0.4299 (3)	0.5211 (6)	0.1118 (8)	0.0506 (15)	
H5	0.4494	0.6067	0.1527	0.061*	
C6	0.4659 (3)	0.4006 (5)	0.1792 (8)	0.0421 (13)	
N1	0.4378 (2)	0.2774 (5)	0.1198 (6)	0.0412 (11)	
Cd1	0.5000	0.07821 (6)	0.2500	0.0476 (2)	
Br1	0.41572 (3)	−0.09792 (6)	0.02140 (9)	0.0507 (2)	

### Atomic displacement parameters (Å^2^)

**Table:**

	*U*^11^	*U*^22^	*U*^33^	*U*^12^	*U*^13^	*U*^23^
C1	0.041 (3)	0.047 (3)	0.054 (4)	−0.004 (3)	−0.001 (3)	0.008 (3)
C2	0.041 (3)	0.066 (4)	0.055 (4)	0.003 (3)	0.008 (3)	0.020 (3)
C3	0.048 (4)	0.109 (6)	0.086 (6)	0.006 (4)	−0.005 (4)	0.034 (5)
C4	0.055 (4)	0.061 (4)	0.069 (4)	0.026 (3)	0.026 (3)	0.029 (4)
C5	0.062 (4)	0.040 (3)	0.056 (4)	0.012 (3)	0.027 (3)	0.007 (3)
C6	0.044 (3)	0.036 (3)	0.049 (3)	0.003 (2)	0.017 (3)	0.007 (2)
N1	0.033 (2)	0.038 (2)	0.049 (3)	0.0021 (18)	0.003 (2)	0.006 (2)
Cd1	0.0456 (4)	0.0318 (3)	0.0530 (4)	0.000	−0.0102 (3)	0.000
Br1	0.0474 (4)	0.0423 (3)	0.0550 (4)	−0.0094 (2)	−0.0006 (3)	−0.0064 (2)

### Geometric parameters (Å, º)

**Table:**

C1—N1	1.339 (6)	C5—C6	1.388 (8)
C1—C2	1.400 (8)	C5—H5	0.9300
C1—H1	0.9300	C6—N1	1.339 (7)
C2—C4	1.366 (10)	C6—C6^i^	1.481 (11)
C2—C3	1.497 (9)	Cd1—N1	2.352 (4)
C3—H3A	0.9600	Cd1—N1^i^	2.352 (4)
C3—H3B	0.9600	Cd1—Br1	2.6676 (8)
C3—H3C	0.9600	Cd1—Br1^i^	2.6676 (8)
C4—C5	1.366 (9)	Cd1—Br1^ii^	2.9351 (10)
C4—H4	0.9300	Cd1—Br1^iii^	2.9352 (10)
			
N1—C1—C2	122.3 (6)	C5—C6—C6^i^	123.0 (4)
N1—C1—H1	118.8	C6—N1—C1	120.0 (5)
C2—C1—H1	118.8	C6—N1—Cd1	117.6 (3)
C4—C2—C1	117.3 (5)	C1—N1—Cd1	122.4 (4)
C4—C2—C3	123.3 (6)	N1—Cd1—N1^i^	70.3 (2)
C1—C2—C3	119.4 (6)	N1—Cd1—Br1	94.81 (10)
C2—C3—H3A	109.5	N1^i^—Cd1—Br1	163.48 (11)
C2—C3—H3B	109.5	N1—Cd1—Br1^i^	163.48 (11)
H3A—C3—H3B	109.5	N1^i^—Cd1—Br1^i^	94.81 (10)
C2—C3—H3C	109.5	Br1—Cd1—Br1^i^	100.78 (4)
H3A—C3—H3C	109.5	N1—Cd1—Br1^ii^	84.78 (12)
H3B—C3—H3C	109.5	N1^i^—Cd1—Br1^ii^	89.13 (12)
C5—C4—C2	120.2 (6)	Br1—Cd1—Br1^ii^	96.79 (3)
C5—C4—H4	119.9	Br1^i^—Cd1—Br1^ii^	87.96 (3)
C2—C4—H4	119.9	N1—Cd1—Br1^iii^	89.13 (12)
C4—C5—C6	120.4 (6)	N1^i^—Cd1—Br1^iii^	84.78 (12)
C4—C5—H5	119.8	Br1—Cd1—Br1^iii^	87.96 (2)
C6—C5—H5	119.8	Br1^i^—Cd1—Br1^iii^	96.79 (3)
N1—C6—C5	119.7 (5)	Br1^ii^—Cd1—Br1^iii^	172.56 (3)
N1—C6—C6^i^	117.3 (3)	Cd1—Br1—Cd1^iii^	92.04 (3)
			
N1—C1—C2—C4	0.5 (10)	C1—N1—Cd1—N1^i^	−177.4 (6)
N1—C1—C2—C3	−178.9 (6)	C6—N1—Cd1—Br1	−172.9 (4)
C1—C2—C4—C5	−0.6 (10)	C1—N1—Cd1—Br1	10.0 (5)
C3—C2—C4—C5	178.8 (6)	C6—N1—Cd1—Br1^i^	26.4 (8)
C2—C4—C5—C6	0.2 (10)	C1—N1—Cd1—Br1^i^	−150.8 (4)
C4—C5—C6—N1	0.2 (10)	C6—N1—Cd1—Br1^ii^	90.7 (4)
C4—C5—C6—C6^i^	−177.8 (7)	C1—N1—Cd1—Br1^ii^	−86.4 (4)
C5—C6—N1—C1	−0.3 (9)	C6—N1—Cd1—Br1^iii^	−85.0 (4)
C6^i^—C6—N1—C1	177.8 (6)	C1—N1—Cd1—Br1^iii^	97.8 (4)
C5—C6—N1—Cd1	−177.5 (4)	N1—Cd1—Br1—Cd1^iii^	88.96 (12)
C6^i^—C6—N1—Cd1	0.5 (9)	N1^i^—Cd1—Br1—Cd1^iii^	63.9 (4)
C2—C1—N1—C6	−0.1 (9)	Br1^i^—Cd1—Br1—Cd1^iii^	−96.53 (2)
C2—C1—N1—Cd1	177.0 (5)	Br1^ii^—Cd1—Br1—Cd1^iii^	174.26 (2)
C6—N1—Cd1—N1^i^	−0.2 (3)	Br1^iii^—Cd1—Br1—Cd1^iii^	0.0

Symmetry codes: (i) −*x*+1, *y*, −*z*+1/2; (ii) *x*, −*y*, *z*+1/2; (iii) −*x*+1, −*y*, −*z*.
